# The Differential Expression of ERAP1/ERAP2 and Immune Cell Activation in Pre-eclampsia

**DOI:** 10.3389/fimmu.2020.00396

**Published:** 2020-03-10

**Authors:** Kimberly Seamon, Lesia O. Kurlak, Michelle Warthan, Efstratios Stratikos, Jerome F. Strauss, Hiten D. Mistry, Eun D. Lee

**Affiliations:** ^1^Virginia Commonwealth University School of Medicine, Richmond, VA, United States; ^2^Division of Child Heath, Obstetrics & Gynaecology, School of Medicine, University of Nottingham, Nottingham, United Kingdom; ^3^University of Virginia, Charlottesville, VA, United States; ^4^National Center for Scientific Research Demokritos, Athens, Greece; ^5^Department of Obstetrics and Gynecology, Virginia Commonwealth University, Richmond, VA, United States; ^6^Department of Microbiology and Immunology, Virginia Commonwealth University, Richmond, VA, United States

**Keywords:** immunology, hypertension in pregnancy, placenta, trophoblast, first trimester

## Abstract

Pre-eclampsia (PE) is a disorder of pregnancy, often leading to serious and fatal complications. Endoplasmic reticulum aminopeptidase 1 and 2 (ERAP1/ERAP2) are present in the placenta. They are involved in processes regulating blood pressure, angiogenesis, cytokine receptor shedding, and immune recognition. Previous studies have associated both ERAP1/ERAP2 genetic variants with PE, although the underlying mechanisms remain unknown. Less is known about the roles for these enzymes in early placentation, which could be a contributory factor to PE. To ascertain whether ERAP1/ERAP2 change in PE and whether such a change is present before PE is clinically diagnosed, we analyzed mRNA and ERAP1/2 protein expression in the placenta in the early first trimester (8–14 weeks) and at delivery in normotensive or PE women (*n* = 12/group). Gene expression was analyzed using qPCR, and protein expression and localization were assessed by immunohistochemistry. Additionally, we profiled peripheral immune cells from normotensive and PE (*n* = 5/group) women for activation and expression of cytotoxic markers using flow cytometry to investigate a possible correlation with placental expression of ERAP1/2. Finally, we characterized the cytokines released from immune cells isolated from normotensive women and those with PE, stimulated *ex vivo* by JEG-3 trophoblast cells. The ERAP1 protein was significantly upregulated in first trimester placentae compared to placentae at delivery from both normotensive and PE women (*p* < 0.05): expression of placental ERAP1 protein was also relatively higher in normotensive than PE women. Although the protein expression of both ERAP1/ERAP2 was significantly lower in women with PE compared to normotensive controls (*p* < 0.05), ERAP2 protein expression remained unchanged in normotensive women at delivery compared to expression in the first trimester. Flow cytometry analysis revealed an increase in activation and cytotoxic natural killer (NK) cells in peripheral blood of PE compared to normotensive women. Intriguingly, there was a notable difference in cytokine release from the activated immune cells when further stimulated by trophoblast cells. The immune cells from PE released elevated expressions of interleukin (IL)-2, IL-4, and most notably, pro-inflammatory IL-13 and IL-17α, inflammatory cytokines tumor necrosis factor (TNF)-α and interferon (IFN)-γ, and granulocyte-macrophage colony-stimulating factor (GM-CSF) compared to normal peripheral blood mononuclear cells (PBMCs). Taken together, these findings suggest that differential lymphocyte activation could be associated with altered ERAP1/ERAP2 expression.

## Introduction

The establishment of a healthy pregnancy requires a finely orchestrated balance of immune cell responses and regulatory secretory mechanisms to prevent excessive systemic inflammation while promoting trophoblast invasion and angiogenesis, leading to placentation.

Endoplasmic reticulum aminopeptidase 1 and 2 (ERAP1/ERAP2) are zinc-metallopeptidases involved in immune recognition, shedding of several cytokine receptors, and other critical processes in pregnancy, including the regulation of blood pressure and angiogenesis. Abnormal placentation and subsequent systemic excessive inflammatory responses are characteristics of pre-eclampsia (PE), which affects 5–8% of pregnancies worldwide, resulting in preterm birth, neonatal fatality, and even maternal death. Previous genetic studies associated both *ERAP1/ERAP2* variants with PE (1, 2), although the exact mechanisms are still unknown, with even less known about their role in placentation.

Clinically, PE is characterized by hypertension (>140/90 mmHg on two separate occasions) after 20-week gestation in a previously normotensive woman, with significant proteinuria (>300 mg/24 h or urinary protein:creatinine ratio >30 mg/mmol) (3). As a multifactorial syndrome, it is significantly influenced by immunological response, vascularization, trophoblast invasion, as well as an underlying genetic predisposition (1, 4). Recognition events that facilitate immune interaction between maternal decidual T cells, uterine natural killer (NK) cells, and cytotrophoblasts may indirectly impact the remodeling of the spiral arteries in PE, while previous studies report increased infiltration of neutrophils into the maternal vasculature (5). Linkage analysis studies, focused on understanding the genetic predisposition of women for developing PE, have identified multiple loci to be significantly associated with this disorder (1). It is important to emphasize that it is the variations in fetal (not maternal) *ERAP2* gene that have been associated with PE by population-based studies in Norwegian, Australian, and African American ethnicities (2, 6). In addition, these studies have also noted that the *ERAP2* gene carries different variants in each ethnic group despite the associated pathophysiological condition being identical.

Immunological functions of ERAP1/ERAP2 include intracellular cleavage and processing of antigenic precursor peptides that are presented by major histocompatibility complex (MHC) class I molecules on the cell surface. This assembly of a cell surface protein carrying a peptide on MHC class I is recognized by CD8-positive cytotoxic T cells and NK cells via their own cell surface receptors. A balanced interaction between the MHC molecules and lymphocytes is essential for triggering an expected immune response. Indeed, it has been shown that both ERAP1 and ERAP2 are able to regulate CD8+ T and NK responses in cultured cells (7, 8).

*In utero*, ERAP2 protein is expressed in feto-placental derived cells that play an active role in implantation and placentation (9). However, comprehensive characterization of both ERAP1 and ERAP2 expression in the placenta from women with PE has not been reported. Since immune responses play such a significant role in the pathophysiology of PE, we speculate that the ERAP2-mediated change in antigen presentation is one of the underlying contributory molecular mechanisms potentially affecting the profile of both uterine and peripheral maternal immune cells. More specifically, an increase in the percentage of T helper (Th)1 cells and ratios of Th1:Th2 cells in women experiencing PE compared to normotensive pregnant women (10). It has been suggested that this shift primarily involves aberrant activation of NK cells in the decidua and maternal blood, thus causing the changes seen in PE (11).

We recently reported that JEG-3 cells expressing the ERAP2 K variant, which is genetically associated with PE, were preferentially targeted by cytotoxic NK cells. We hypothesize that the constant exposure to feto-placental antigen alters the uterine immune environment, priming the peripheral immune cells, and thus revealing the early activation markers of cytotoxic pNK and T cells. The aims of this study were first to determine placental expression patterns of ERAP1/ERAP2 early in pregnancy (first trimester) and at delivery in normotensive controls and women with PE. Second, to describe and compare peripheral NK (pNK) cell and T cell populations in normotensive women and those with PE to characterize markers of lymphocyte activation and binding capability as a result of the constant exposure to ERAP1/ERAP2-generated fetal peptides. Third, to determine the cytokine profile produced by activated immune cells against ERAP1/ERAP2-expressing trophoblasts.

## Results

The demographic and obstetrical data of study participants are shown in Table 1. Within the group of women who had PE, six had early onset (diagnosis before 34 weeks' gestation) and six had late onset (diagnosis after 34 weeks' gestation). All women delivered singleton babies.

**Table 1:** Demographic, clinical, and biochemical data of participants.

**Parameter**	**First trimester (*n* = 12)**	**Normotensive (*n* = 12)**	**Pre-eclampsia (*n* = 12)**
Maternal age (years)	–	32 ± 6.1	30 ± 7.9
Booking body mass index (kg/m^2^)	–	26.5 ± 4.8	29.7 ± 6.8
Maximum systolic blood pressure outside labor (mmHg)	–	129 ± 6.1	153 ± 9.2^**^
Maximum diastolic blood pressure outside labor (mmHg)	–	83 ± 6.4	99 ± 5.2^**^
Protein:creatinine ratio (g/mmol)	–	–	192 (94, 262)
Gestational age at delivery (weeks)	9.0 ± 1.5	39.2 ± 0.5	36.4 ± 2.7^*^
Cesarean section [No. (%)]	–	8 (67)	10 (83)
Number of male babies [No. (%)]	–	4 (33)	8 (67)
Birth weight (kg)	–	3.65 (3.37, 3.94)	2.55 (2.01, 3.06)^*^

* P < 0.05;

** *P < 0.0001 between normotensive and pre-eclampsia diagnostic groups*.

### Placental ERAP1/ERAP2 mRNA and Protein Expression

**Figure 1:**
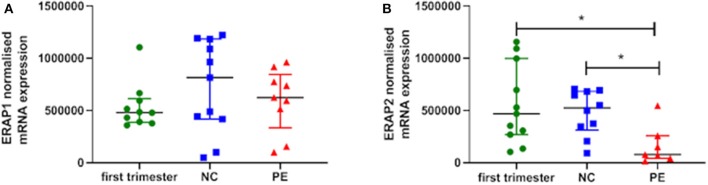
Normalized mRNA expression (copy number) of **(A)** endoplasmic reticulum aminopeptidase (ERAP)1 and **(B)** ERAP2 in placentae from first trimester (*n* = 12), term normotensive controls (NC; *n* = 12), and women with pre-eclampsia (PE; *n* = 12). Data are presented as median [interquartile range (IQR)]; *P < 0.05.

*ERAP1* and *ERAP2* mRNA expression was detected in placental tissue from all gestations. No significant differences were observed for *ERAP1* between groups (*P* > 0.05; Figure 1A). However, when considering *ERAP2*, placentae from women with PE had the lowest expression {median [interquartile range (IQR)], normalized copy number: 0.78 × 10^5^ [5.4 × 10^4^, 2.1 × 10^6^]}, which were significantly reduced compared to both the first trimester placentae [4.7 × 10^5^ (2.9 × 10^5^, 8.5 × 10^5^); *P* = 0.01] and normotensive controls [5.3 × 10^5^ (3.5 × 10^5^, 6.8 × 10^5^); *P* = 0.007] placentae (Figure 1B).

**Figure 2:**
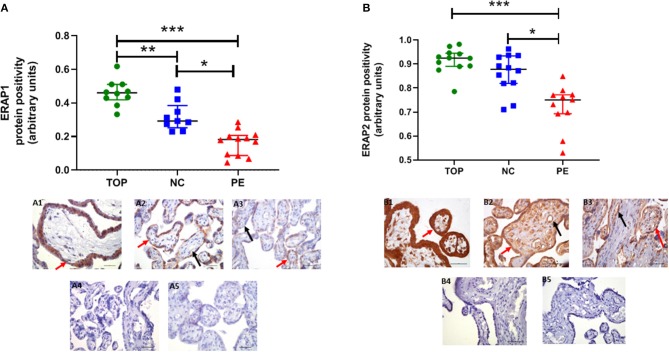
Protein expression assessed by immunohistochemistry. **(A)** Endoplasmic reticulum aminopeptidase (ERAP)1, **(B)** ERAP2 in placentae from first trimester (TOP; *n* = 12), term normotensive controls (NC; *n* = 12), and women with pre-eclampsia (PE; *n* = 12). Data are presented as median [interquartile range (IQR)]; *P < 0.05; **P < 0.01; ***P < 0.0001. Photomicrographs show typical examples of immunostaining in (A1) TOP, (A2) NC, (A3) PE, (A4) IgG negative control, (A5) negative control—no primary antibody. Positive protein expression appears brown and is localized mainly to the syncytiotrophoblast (red arrows) but is also evident around the fetal vessels (black arrows); scale bar = 100 μm; magnification ×400.

**Figure 3:**
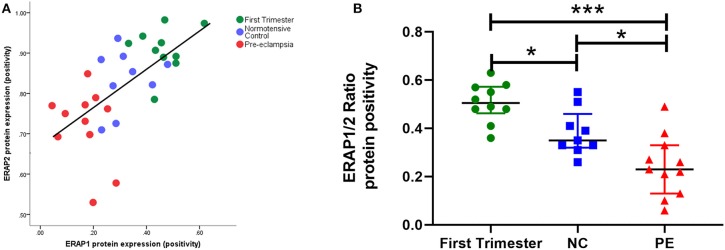
**(A)** Scatter plot illustration shows a strong positive correlation between endoplasmic reticulum aminopeptidase (ERAP)1 and ERAP2 placental protein expression (*r* = 0.70; *P* < 0.0001); **(B)** box plots of ERAP1/ERAP2 ratios in first trimester tissue (*n* = 12), normotensive control (NC; *n* = 12), and pre-eclampsia (PE; *n* = 12). Data are presented as median [interquartile range (IQR)]; *P < 0.05; ***P < 0.0001.

Using immunohistochemistry, ERAP1 and ERAP2 proteins were localized in syncytiotrophoblasts and fetal vessels (Figures 2A,B). The highest expression of both proteins was detected in the first trimester. ERAP1 protein expression was increased in the first trimester [median (IQR) positivity: 0.46 (0.43, 0.51)] compared to both normotensive controls [0.29 (0.27, 0.35)] and PE [0.17 (0.10, 0.20)] samples (*P* < 0.0001; Figure 2A). For ERAP2, placental [0.92 (0.89, 0.94)] expression was only significantly higher in the first trimester compared to PE placental expression [0.75 (0.70, 0.77); *P* < 0.0001], but not normotensive controls [0.88 (0.82, 0.93); *P* > 0.05; Figure 2B]. However, in PE, the expression of both ERAP1/ERAP2 was lower compared to that in normotensive controls (ERAP1: *P* < 0.0001; ERAP2: *P* = 0.001; Figure 2B). Overall, there was a strong correlation between placental ERAP1 and ERAP2 expression (*r* = 0.70; *P* < 0.0001; Figure 3A).

When considering ratios of protein expression, ERAP1/ERAP2 ratios were significantly different between groups (*P* < 0.0001). The highest ratios were in early pregnancy [0.51 (0.048, 0.57)]; normotensive [0.35 (0.33, 0.41); *P* = 0.01 compared to first trimester]; PE [0.23 (0.7, 0.30); *P* < 0.0001 compared to first trimester]. Furthermore, the placentae from PE women had significantly lower ratios compared to their normotensive counterparts (*P* = 0.01; Figure 3B).

Gestational age at delivery was significantly lower in the women with PE (Table 1); therefore, to ensure that the observed differences were not related to gestational age, we also compared the data with controls only for the PE women who delivered ≥37 weeks' gestation. All comparisons remained statistically different.

### Immune Populations in Normotensive Controls and Pre-eclampsia Peripheral Blood

**Figure 4:**
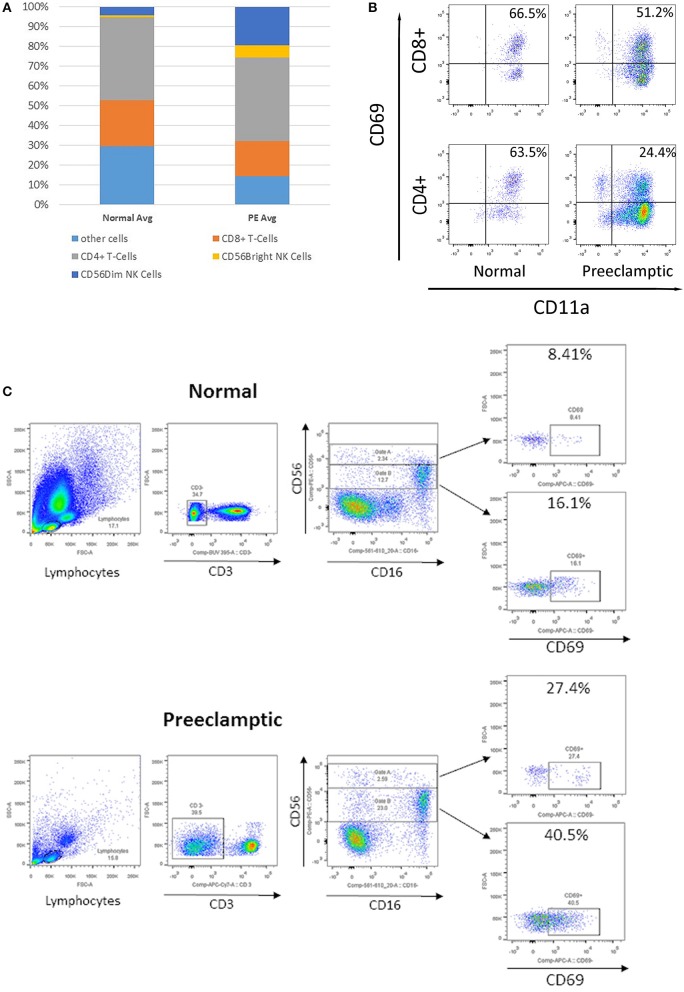
Flow cytometry analysis of peripheral immune cells in blood from normotensive control and pre-eclampsia (PE) women taken before delivery. **(A)** The relative proportion of T cells and natural killer (NK) cells. **(B)** Flow cytometry analysis of CD69+, CD11a+, CD8+, and CD4+ T cells. **(C)** Analysis of CD3–, CD56+, and CD16+ NK cells, divided into CD56 Bright (Gate A) and Dim NK cells (Gate B), and early activation CD69+.

The complete lymphocyte profile was characterized using flow cytometry (Figure 4A). In peripheral blood from women with PE, the percentage of total CD4+ and CD8+ T cells remain similar, whereas the percentage of CD56+ NK cells was increased. Within the PE population, CD56^Bright^ NK cells were similar but increased compared to normotensive women. When NK and T cells were more comprehensively defined, there was a three-fold decrease in CD4+, CD69+, and CD11a+ T cells in PE compared to normotensive women (24.4 vs. 63.5%; Figure 4B). However, only a slight decrease in CD69+, CD11a+, and CD8+ T cells was detected in PE women compared to normotensive controls (51.2 vs. 66.5%). Interestingly, the abundance of activated CD69+ in both CD56+Dim and CD56+Bright NK cells was higher in PE (Bright: 8.4 vs. 27.4% Dim: 16.1 vs. 40.5%; Figure 4C). Subsequently, the CD56+ NK cells were differentiated into Dim/Bright ratio, with CD16 to distinguish the cytotoxicity. Most importantly, the ratio of activated NK cells characterized by CD69+/CD56^Dim^/CD16+, where the cytotoxicity of this population has been demonstrated in a previous *in vitro* study, was almost doubled in PE (12.7 vs. 23%; Figure 4C) (12). This suggests that activated and more cytotoxic NK cells are circulating in women with PE.

### Cytokine Profile in Supernatants of Peripheral Blood Mononuclear Cells Following Stimulation With JEG-3 Cells

To reveal if normotensive control and PE maternal peripheral blood have different cytokine profiles due to varying immune phenotypes and responses, cytokine antibody array analysis was performed with the supernatants from PBMCs exposed to trophoblast-derived JEG-3 cell lines (Table 2). The activation assay revealed differences in both the extent of expression and dissimilar patterns of a number of Th1, Th2, and Th17 cytokines. The lymphocytes from normotensive controls and PE, stimulated by the ERAP1/ERAP2-expressing JEG-3 cells, released the same levels of interleukin (IL)-6 and IL-8. On the other hand, the lymphocytes from PE women had higher expression of IL-2, IL-4, IL-10, IL-13, IL-17α, and, most notably, in stimulatory factors such as tumor necrosis factor (TNF)-α, granulocyte-macrophage colony-stimulating factor (GM-CSF), and interferon (IFN)-γ when compared to the expression of other cytokines.

**Table 2:** Analysis of the cytokine profile in the supernatants of lymphocyte cell cultures derived from peripheral blood mononuclear cells (PBMCs) from normotensive control and pre-eclamptic women (PE), stimulated with endoplasmic reticulum aminopeptidase (ERAP)1/ERAP2-expressing JEG-3 trophoblasts.

**Cytokine**	**JEG-3/Normal**	**JEG-3/PE**
IL-2	–	++
IL-4	–	++
IL-5	–	–
IL-6	+++	+++
IL-8	+++	+++
IL-10	++	+++
IL-12p70	–	–
IL-13	–	+
IL-17a	–	++
TNF-α	++	+++
GM-CSF	+	++
IFN-γ	+	++

*(–) No detection, (+) low detection, (++) medium detection, and (+++) strong detection. GM-CSF, granulocyte-macrophage colony-stimulating factor; IFN, interferon; IL, interleukin; TNF-tumor necrosis factor*.

## Discussion

This novel study establishes the presence of ERAP1/ERAP2 gene and protein expression in placentae from the first trimester as well as in the third trimester, at delivery; it is also the first to compare the expression in women who had a normotensive pregnancy and those who had PE. Our results revealed temporal differences in expression level, as well as lower expression in PE. Furthermore, we report a novel observation that peripheral activated NK cell populations are increased in PE. We speculate that this could be a mechanism to maintain an immune balance. In addition, the differential profile of peripheral immune cells and the cytokines released from them in response to JEG-3 cells transfected with ERAP2K variant suggests a possible molecular role in placentation.

The roles of ERAP enzymes in biological processes include regulation of immune responses, angiogenesis, and blood pressure regulation (13), all of which have been implicated in placental function and hence PE. The lower ERAP1 protein expression observed in PE placentae could contribute to a shift in the balance between activating and inhibitory signals toward NK cell activation. This could result in target cell killing, as has been reported in murine T cell lymphoma model following a reduction of ERAP1 (13). The current study shows that in humans, lower ERAP1 and constant exposure to ERAP2 may play a role in elevating the numbers of peripheral activated cytotoxic NK cells in PE. The constant circulation of highly activated NK cells could account for some of the PE symptoms caused by a dysregulated innate immune response and may potentially explain why blood from normotensive and PE women responded differently to ERAP1/ERAP2-expressing trophoblast cells. We speculated that the presence of ERAP2 may be unfavorable as early as during placentation and throughout pregnancy if the balance of ERAP1/ERAP2 expression is disturbed, which may result in an imbalance of NK cells. A future study is required to test the hypothesis that ERAP2 may potentially be destroying epitopes that results in major histocompatibility complex (MHC) that carry suboptimal peptides, which can fail to be recognized by inhibitory killer cell immunoglobulin-like receptor (KIR) or recognized by activating KIR on NK cells.

Additional differences in normotensive and PE peripheral blood are observed in the cytokine profile, which indicates that PBMCs from healthy pregnancies do not release lymphocyte activating IL-2, which limits the activation and proliferation of lymphocytes. In contrast, PBMCs from women with PE show elevated expressions of IL-2, IL-4, and most notably, pro-inflammatory IL-13, IL-17a, and inflammatory TNF-α, GM-CSF, and IFN-γ against JEG-3 cells.

The crucial balance level of ERAP1/ERAP2 protein expression in first trimester placentae suggests a beneficial function of these enzymes in early placentation due to their multifunctional roles in proliferation angiogenesis, as well as associations with the renin-angiotensin system (RAS), with a high expression of the angiotensin II (Ang II) receptors also present in first trimester placentae (14). Further studies are required to elucidate fully their specific roles in early placentation and extravillous trophoblast invasion.

Moreover, the lower placental expression of ERAP1 and higher expression level of ERAP2 early than at term in PE may also contribute to the regulation of the RAS due to their known role in cleaving Ang II and Ang III (15), thus contributing to the regulation of blood pressure. A chaperone protein found in the endoplasmic reticulum, ERp44, is known to regulate Ang II by forming a mixed disulfide bond with ERAP1, and secreted ERAP1 promotes hypotension in an animal model (16). Moreover, ERp44 placental expression has been reported to be six times higher in PE compared to placentae from normotensive pregnancies (17). Therefore, we speculate that in PE, the higher ERp44 forms a complex with ERAP1, preventing the secretion and cleavage of Ang II, which would otherwise promote hypertension. We have previously reported that in PE, there is an increased placental expression of the type 1 receptor for Ang II (AT1R) and reduced AT4R, the specific receptor for Angiotensin 3–8 (AngIV), which counteracts the hypertensive effects mediated by AT1R (14, 18). Thus, we suggest that this, in combination with the higher ERp44-ERAP1 complex, may contribute to the increased blood pressure so characteristic of PE, although further work is clearly required to confirm this.

## Materials and Methods

### Subject and Selection Criteria

All the participants in the study were white women of European ancestry: 12 undergoing termination of pregnancy; 12 normotensives, 12 with PE (Table 1), with paired placental samples and maternal blood collected. PE was defined as systolic blood pressure of ≥140/90 mmHg on two occasions at least 6 h apart and proteinuria ≥300 mg/24 h, or urinary protein:creatinine ratio >30 mg/mmol or ≥2+ on a dipstick analysis of midstream urine, after 20 weeks of gestation (3). All women who took part in this study were laboring and either delivered vaginally or by emergency Cesarean section; no differences were observed in any measurements between Cesarean section and vaginal deliveries.

### Sample Collection

Placental tissue was dissected from products of conception obtained from women undergoing elective surgical termination of pregnancy between 8 and 14 weeks (mean ± SD gestational age 9.0 ± 1.5 weeks). For comparison between normotensive women and those with PE, maternal venous blood samples were taken prior to delivery. All blood samples were collected in EDTA anticoagulant (plasma) or plain tubes (serum). Full-depth tissue biopsies were collected within 10 min of the placenta being delivered for the near term samples, as previously described (19), sampling halfway between the cord insertion and periphery of the placenta, avoiding infarcts.

### RNA Extraction, cDNA Synthesis, and Quantitative Real-Time PCR

Total RNA was extracted from ~100 mg placental tissue using QIAzol lysis reagent (Qiagen, UK), as previously described (20). RNA (1 μg) was reverse transcribed using the QuantiTect reverse transcription kit (Qiagen) in a Primus96 thermocycler (Peqlab Ltd., UK). Real-time PCR was carried out using SYBR Green chemistry (Fast SYBR™ Green Master Mix; Applied Biosystems, USA) on an AB7500 Fast (Life Technologies, UK) using the primers in Table 3. Abundance data for the genes of interest were expressed following normalization using GeNORM (https://genorm.cmgg.be/), with stably expressed reference genes [glyceraldehyde 3-phosphate dehydrogenase (GAPDH), β-2-microglobulin, and β-actin] expressed as normalized copy number (21).

**Table 3:** Primer details.

**Gene**	**Accession number**	**Primers**	**Length (bp)**
ERAP1	NM_005534	5′-ctcctcagcacccgaagattc-3′	152
		5′-gccgtgaaccatttactgtcg-3′	
ERAP2	NM_022350	5′-ccagagaaacttacgcctcac-3′	155
		5′-gcctgggttggctcaaaatc-3′	

*ERAP, endoplasmic reticulum aminopeptidase*.

### Immunohistochemical Staining

Placental protein expression was assessed by immunohistochemistry, as previously described (22), using paraffin-embedded tissue sections (5 μm). Protein expression of ERAP1/ERAP2 using anti-ARTS1/ERAP1 rabbit monoclonal (ab232466; dilution 1:50; Abcam) and anti-LRAP/ERAP2 goat polyclonal (AF3830; 8 μg/ml; R&D Systems) antibodies were used. All of the slides were assessed by the same observer, blinded to pregnancy outcome. For analysis of placental sections, digital images of five randomly selected, high-power (×400 magnification) fields were captured on NIS-Elements F2.20 microscope (Nikon United Kingdom Ltd., Surrey, UK). Quantification of protein expression was performed as described previously (14) using the Positive Pixel Algorithm of Aperio ImageScope software. This software is able to discriminate between positive- and negative-stained pixels and combines the number of positive pixels stained with the intensity of these same pixels to produce the value “positivity.” The positivity is expressed as a percentage of total pixels detected and thus takes into account tissue area. A visual check was also performed to ensure accurate discrimination of immunolabeled regions.

### Immune Populations in Normotensive Controls and Pre-eclampsia by Flow Cytometry

Collection and preparation were previously described (12). Red blood cells were lysed using ice-cold deionized water for 30 s. White blood cells were then resuspended in FACs buffer [1× phosphate buffered saline (PBS), 2% heat-inactivated fetal bovine serum (FBS)], counted, and blocked for 10 min in FACS Buffer and Human Trustain FcX Fc receptor blocking solution (Biolegend; #422302) at room temperature, followed by staining with CD3-APC/Fire750 (BioLegend; #344840), CD56-PE (BioLegend; #318306), CD8-PerCP (BioLegend; #344708), CD11a-PE/Cy7 (BioLegend; #301220), CD16-FITC (BioLegend; #302006), CD69-APC (BioLegend; #310910). The corresponding immunoglobulin G (IgG) isotype controls were used for staining the lymphocytes. Cells were analyzed on a BD FACSCanto™ II, and the FACs data were analyzed using FlowJo V.10 data analysis software.

### Cytokine Profile in Supernatants of Lymphocytes, Following Stimulation With JEG-3 Cells

JEG-3 cells were transfected with ERAP2K as previously described (12) prior to the addition of the isolated PMBCs. After a 24-h incubation period, the cytokine profile of the collected supernatant was determined using the PathScan® Th1/Th2/Th17 Cytokine Antibody Array Kit according to the manufacturer's protocol (Cell Signaling Technology; #13047).

### Statistical Analysis

All tests were performed using SPSS for Windows version 24 and GraphPad Prism, version 9. The Kruskal–Wallis test, followed by Mann–Whitney U-test was used for multiple group analysis. The Student's *t*-test or Mann–Whitney U-tests were used according to the distribution of the data, as assessed by the Kolmogorov–Smirnov test. Spearman's rank correlations tests were used to explore associations between continuous variables. The null hypothesis was rejected where *P* < 0.05.

## Data Availability Statement

All datasets generated for this study are included in the article/supplementary material.

## Ethics Statement

Each study participant gave written informed consent before inclusion in the study, which was approved by the Nottingham Hospitals Trust Ethics Committee (15/EM/0523) and Virginia Commonwealth University IRB (HM20001364). The study wasconducted in accordance with the Declaration of Helsinski.

## Author Contributions

HM, LK, KS, and EL performed the experiments, analyzed the data, and wrote the manuscript. KS performed and analyzed the AFM experiments. HM, LK, and EL participated to the study design, contributed to the manuscript, and supervised the study. HM, LK, EL, and MW conceived the study and recruited samples. ES and JS read and revised the manuscript.

### Conflict of Interest

The authors declare that the research was conducted in the absence of any commercial or financial relationships that could be construed as a potential conflict of interest.
